# Galectin-9: A Suppressor of Atherosclerosis?

**DOI:** 10.3389/fimmu.2020.604265

**Published:** 2020-11-04

**Authors:** Jian Yu, Ruirui Zhu, Kunwu Yu, Yue Wang, Yan Ding, Yucheng Zhong, Qiutang Zeng

**Affiliations:** Department of Cardiology, Union Hospital, Tongji Medical College, Huazhong University of Science and Technology, Wuhan, China

**Keywords:** Galectin-9, atherosclerosis, T-cell immunoglobulin mucin 3, regulatory T cells, T helper cells

## Abstract

It is no longer controversial that atherosclerosis is a vascular wall chronic inflammatory disease mediated by cells of innate and adaptive immunity. Galectin-9 (Gal-9) seems to be a crucial regulator of T-cell immunity by inducing apoptosis in specific T-cell subpopulations associated with autoimmunity and inflammatory disease. Accumulating evidence showed that galectin-9 signaling *via* T-cell immunoglobulin mucin 3 (TIM-3) is concerned with different regulatory functions in autoimmunity, including direct depletion of pro-inflammatory T-cells, expanding the number of regulatory T cells, altering macrophages to an anti-inflammatory state and the induction of repressive myeloid-derived suppressor cells. In addition, anti-Tim-3-Ab administration increased atherosclerotic plaque formation by blocking Tim-3–galectin-9 interaction. Hence, we hypothesize that galectin-9 may be a novel therapy for atherosclerotic disease. Further researches are needed to investigate the precise effect of galectin-9 in the process of atherosclerosis.

## Introduction

Firstly discovered as a potent eosinophil chemoattractant (1), Galectin-9 (Gal-9) is recently regarded as a multifaceted immune regulator that affects a host of cell types. Gal-9 seems widely distributed in liver, small intestine, thymus, kidney, spleen, lung, cardiac and skeletal muscle (2). Study results revealed that gal-9 signaling is concerned with regulatory functions in autoimmunity *via* T-cell immunoglobulin mucin 3 (TIM-3) or other yet to be identified receptors, accompanied by altering macrophages to an anti-inflammatory, down-regulating the number of effector T cells and increasing the number of regulatory T cells (Tregs) (3–5). Atherosclerosis is an autoimmune and inflammatory disease. It is reasonable to assume that gal-9 may be involved in atherosclerosis.

### Imbalance of Effector T Cells/Regulatory T Cells in Atherosclerosis

**Figure 1:**
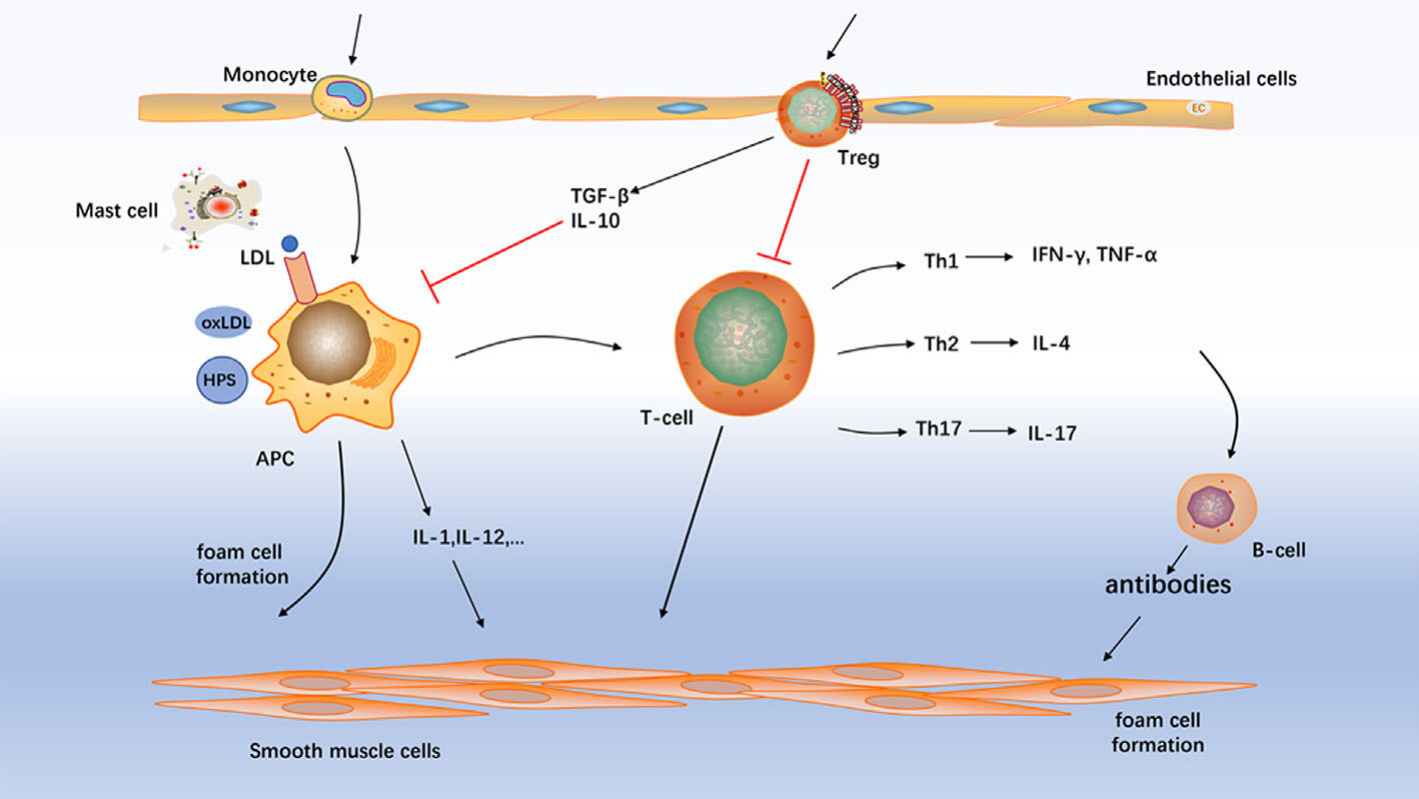
Atherosclerosis mechanism. Several types of immunoreactive cells are present throughout atherosclerosis formation including macrophages, T cells, B cell, and mast cells. Treg, regulatory T-cell; APC, antigen-presenting cell; NK cell, natural killer cells.

Atherosclerosis is a chronic progressive disease of arteries, which is the basis of many important adverse vascular events and the main cause of cardiovascular disease morbidity and mortality. Several types of immunoreactive cells are present throughout atherosclerosis formation including macrophages, T cells, B cells, mast cells and dendritic cells (**Figure 1**). Despite the macrophages are the key cells in atherosclerosis (6), T cells are shown to be concerned with atherogenesis and especially T-cell subpopulations are suggested to trigger/dampen atherosclerotic inflammatory processes (7). Previous studies have showed that the pathogenesis of atherosclerosis is characterized by an imbalance between T-helper 1 (Th1) - and Th2-mediated immune functions (8). Researches have demonstrated Th1 cells to be associated with atherogenesis (9, 10). Furthermore, most of pathogenic T cells in atherosclerosis are of the Th1 profile, which secretes pro-inflammatory cytokines such as interferon- (IFN-) γ and activates monocytes/macrophages and dendritic cells (11). Th1-mediated immune responses contribute to the development of atherosclerosis. In contrast, Th2-biased responses antagonize proatherogenic Th1 functions and thereby confer atheroprotection. Indeed, the role of the Th2 cells in the atherosclerotic process remains controversial and depends on the stage and site of the atherosclerotic lesion (7, 12). The vast majority of studies have shown interleukin- (IL-) 17 contributed to the proinflammatory milieu of atherosclerosis (13–15). However, the role of Th17 cells in atherosclerosis is also controversial. Other studies have shown that Th17 is anti-atherosclerosis (16, 17). Furthermore, we also found that of oxidized low-density lipoprotein inhibited atherosclerosis process *via* decreasing Th17 cell number (18).

In addition to Th1, Th2, and Th17 lineage, CD4+ T cells also include a special subset with anti-atherosclerotic properties, the regulatory T cells. They control the effector functions of activated T cells. Apart from the termination of immune responses, Tregs play a prominent role in inducing and maintaining immunologic tolerance. Recently, studies have shown that activated Treg cells inhibit the process of atherosclerotic disease, and Forkhead box protein P3 (FOXP3) expression controls the transcription program that plays a protective role in atherosclerotic plaques (19). Furthermore, the number of Tregs are decreased in the peripheral blood of patients which suffer from stable coronary artery disease (20). Indeed, our laboratory has showed that Th17/Tregs functional imbalance existed during atherogenesis, indicating that the imbalance of Th17/Tregs plays a pivotal role in atherosclerotic disease (21).

## Galectin-9 as an Immune Regulator

Galectins are β-galactoside binding lectins, which include a highly conserved sequence motif in their carbohydrate recognition domain (22, 23). For all that the entire galectins bind galactose, they have diverse affinity to oligosaccharides (24). Galectins were demonstrated to modulate various cellular functions mainly associated with inflammatory processes, including cell growth, apoptosis, cell adhesion, migration, and immune responses (25). Galectin-9, which was first identified as a potent eosinophil chemoattractant, is currently known as a versatile immunomodulator (26, 27). Notably, up-regulated levels of gal-9 were reported in inflammatory bowel disease (27) and systemic lupus erythematosus (28, 29). The role of gal-9 in immunoregulation seems to be complicated. According to the cellular localization of the respective binding partner, the activity of galectin-9 may be contradictory, intracellular Gal-9 induces proinflammatory cytokine expression in monocytes by forming a complex with transcription factor NF‐IL6 (30), whereas extracellular Gal-9 induces monocyte cell death (31). Originally, gal-9 was found to induce the death of Th1 lymphocytes by the Tim-3 (32). However, it was showed that gal-9 activity was incompletely abrogated by an antagonistic Tim-3 antibody in T-cell lines (33). Interestingly, gal-9 also induced Tim-3 independent apoptosis in both Th1 and Th2 cells at higher concentrations (34). Moreover, gal-9 was found to induce Tim-3 independent production of IFN-γ and Tumor necrosis factor- (TNF-) α in Th1 and Th2 cells at low concentrations, respectively (34). Hence, one or more alternative receptors for gal-9 may exist on T-cells. On the other hand, gal-9 was revealed to induce differentiation of naïve T cells into Tregs, repress the differentiation of Th17 cells *in vitro* and to down-regulate the levels of IL-17 dose-dependently in experimental autoimmune arthritis *in vivo* (35). However, gal-9 induces monocytes migration and significantly higher inflammation in the inflammatory arthritis (36). It may be because the effects of gal-9 vary depending upon the route of administration, concentration and the type of inflammatory model used. Recently, another study also showed that the level of Tim-3 in Th1 cells reduced in patients with maculopapular exanthema and the exogenous recombinant Gal-9 significantly increased Treg proliferation and decreased Th1 proliferation, not differences in the proliferation of Th1 cells after blocking Tim-3 (37). It is certified that Tregs play a prominent role in terminating the immune response. Moreover, Foks et al has revealed that blocking gal-9-Tim-3 interactions can increase the number of macrophages in the atherosclerotic plaque (38). Furthermore, another recent study found that TIM-3 down-regulated the expression of proinflammatory factors by inhibiting the NF-κB signaling pathway and decreased the proliferation and migration of vascular smooth muscle cells, which suggested that TIM-3 showed anti-atherosclerotic effects (39).

## Galectin-9 In Atherosclerosis

It is no longer controversial that an imbalance between the inflammatory immunity and weakened suppressive/regulatory immune response plays a major role in the development of atherosclerosis. Gal-9, the ligand of Tim-3, affects the function of several immune cells such as monocytes, effector T cells, and macrophages, especially regulatory T cells, which are proven involved in atherosclerosis. Gal-9 promoted the differentiation of naïve T-cells into Tregs by enhancing Foxp3 expression, the hallmark transcription factor responsible for Treg differentiation (35). This finding is supported by the research that the number of Tregs is reduced in the gal-9 knock-out mice (35, 40). Gal-9 has been demonstrated to induce apoptosis of Th1 and Th17 cells, recombinant gal-9 may be of potential benefit to restrict acute and chronic autoimmunity. Notably, it was showed that Tregs also express gal-9 on the cell surface (41), which may enhance the suppressive activity of these cells toward Th1 and Th17 cells. Besides, gal-9 influences innate responses in autoimmunity (3). Application of gal-9 suppressed macrophage activity in murine and human immune complex-induced arthritis (3). These immunosuppressive macrophages can also be activated by application of recombinant gal-9, as elucidated in mice with experimental lung inflammation (42). On the other hand, the expression of human gal-9 in transgenic mice led to the differentiation and activation of immunosuppressive granulocytes (CD11b1Ly-6G1) from the bone marrow (5). These immunosuppressive granulocytes, which are termed myeloid-derived suppressor cells (MDSCs), inhibit T-cell immune responses by depleting T-cells and simultaneously expanding the number of Tregs (4, 5). Recently, our research found that the serum level of Gal-9 decreased significantly in the serum of patients with coronary artery disease. Furthermore, the addition of exogenous Gal-9 expanded Tregs and suppressed Th17, leading to increase secretion of transforming growth factor- (TGF-) *β*1 and decrease IL-17 production (43). Interestingly, we further observed an obvious decrease of the relative mRNA expression levels of Gal-9, TIM-3, and Foxp3 in peripheral blood mononuclear cells in patients with acute coronary syndrome (44). Based on these studies, we hypothesized that gal-9 has a significant immunoregulatory function in the process of atherosclerosis and enhancing gal-9 signaling attenuates atherosclerotic plaque development, which is accompanied by decreased monocytes/macrophages and effector T cells and by increased Tregs. This hypothesis is supported by Foks et al. who have demonstrated that anti-Tim-3-Ab administration increased atherosclerotic plaque formation and Tim-3, the receptor of gal-9, acted as a negative regulator of atherosclerosis (38).

**Figure 2:**
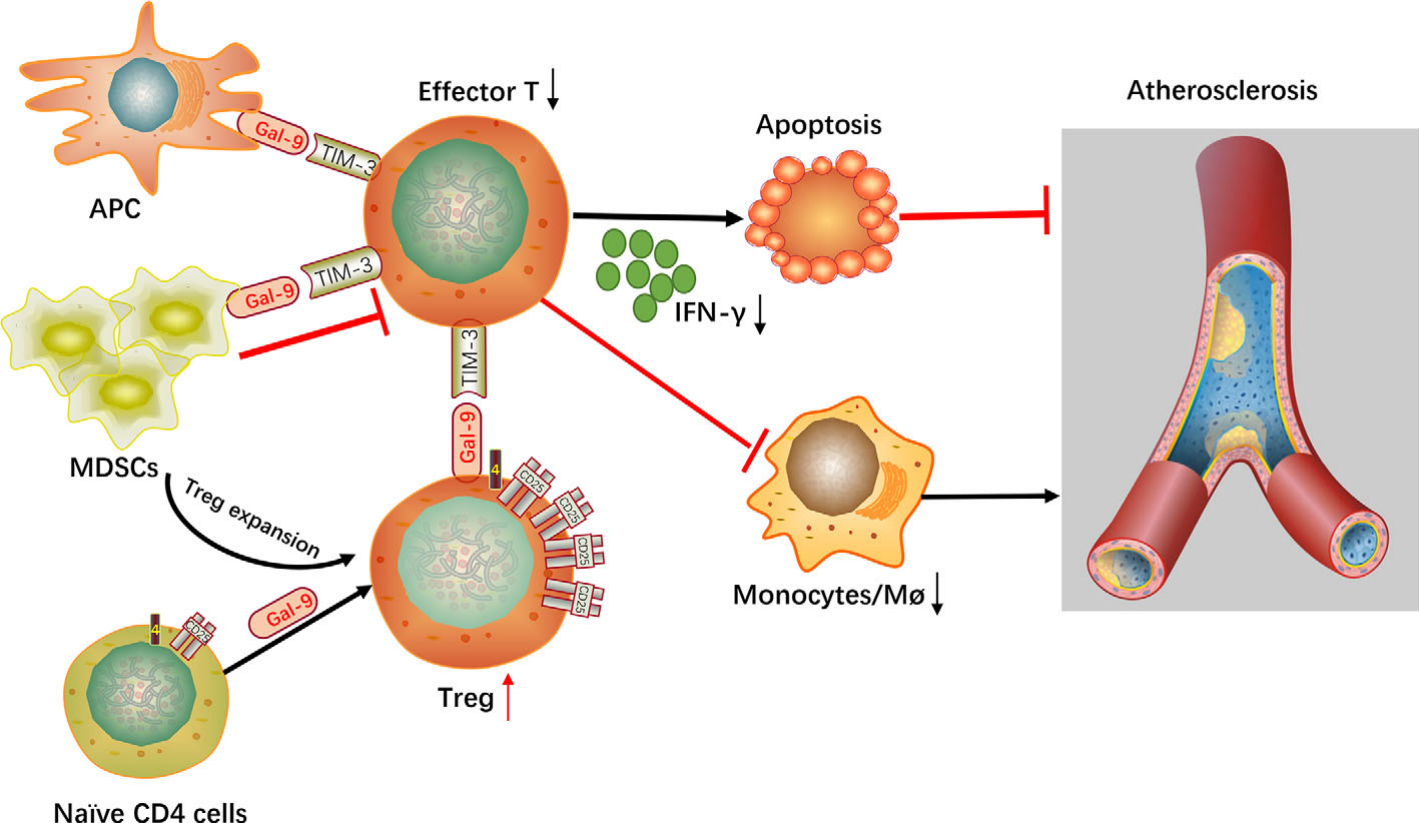
Mechanistic summary. Gal-9, galectin-9; Tim-3, T-cell immunoglobulin mucin 3; Effector T, effector T cells; Treg, regulatory T cell; Mø, macrophages; APC, antigen-presenting cell; MDSCs, myeloid-derived suppressor cells.

In summary, gal-9 signaling *via* TIM-3 or other yet to be determined receptors is concerned with different regulatory functions in autoimmunity, including direct depletion of proinflammatory T-cells, expanding the number of Tregs, altering macrophages to an anti-inflammatory state and the induction of repressive MDSCs. It is particularly noteworthy that blocking gal-9-Tim-3 interactions significantly enhance the development of atherosclerosis and down-regulate Treg cell numbers and up-regulate the numbers of monocytes/macrophages and effector T cells (**Figure 2**). According to these findings, it is very possible that gal-9 will be regarded as a novel therapy for atherosclerotic disease in the near future.

## Author Contributions

JY and RZ wrote the manuscript. QZ and YZ edited the manuscript. KY finished the figures. YW and YD provided the feedback and guidance. All authors contributed to the article and approved the submitted version.

## Funding

This work was supported by grants from the National Natural Science Foundations of China (NO. 81770273, 81900400, 81900270).

## Conflict of Interest

The authors declare that the research was conducted in the absence of any commercial or financial relationships that could be construed as a potential conflict of interest.

## Abbreviations

Gal-9, Galectin-9; FOXP3, forkhead box protein P3; IFN-γ, interferon-γ; IL, interleukin; MDSCs, myeloid-derived suppressor cells; TGF-*β*1, transforming growth factor-β1; Th, T-helper; Tim-3, T-cell immunoglobulin mucin 3; TNF-α, tumor necrosis factor-α; Treg, regulatory T cell.
