# Corrigendum: Galectin-9: A Suppressor of Atherosclerosis?

**DOI:** 10.3389/fimmu.2021.686592

**Published:** 2021-04-13

**Authors:** Jian Yu, Ruirui Zhu, Kunwu Yu, Yue Wang, Yan Ding, Yucheng Zhong, Qiutang Zeng

**Affiliations:** Department of Cardiology, Union Hospital, Tongji Medical College, Huazhong University of Science and Technology, Wuhan, China

**Keywords:** Galectin-9, atherosclerosis, T-cell immunoglobulin mucin 3, regulatory T cells, T helper cells

In the original article, an author name was incorrectly spelled as Kuwu Yu. The correct spelling is Kunwu Yu.

The authors apologize for this error and state that this does not change the scientific conclusions of the article in any way. The original article has been updated.

